# Dual Nature of Neutrophil Extracellular Traps (NETs)—From Cancer’s Ally to Therapeutic Target

**DOI:** 10.3390/cells14151200

**Published:** 2025-08-05

**Authors:** Karolina Buszka, Claudia Dompe, Kinga Derwich, Izabela Pieścikowska, Michał Nowicki, Joanna Budna-Tukan

**Affiliations:** 1 Department of Histology and Embryology, Poznan University of Medical Sciences, 60-781 Poznan, Poland; karolina.buszka@student.ump.edu.pl (K.B.); ipiescikowska@ump.edu.pl (I.P.); mnowicki@ump.edu.pl (M.N.); 2 Doctoral School, Poznan University of Medical Sciences, 60-812 Poznan, Poland; 84416@student.ump.edu.pl; 3 Department of Immunology, Poznan University of Medical Sciences, 60-806 Poznan, Poland; kingaderwich@ump.edu.pl; 4 Department of Anatomy and Histology, Collegium Medicum, University of Zielona Gora, 65-046 Zielona Gora, Poland

**Keywords:** neutrophils, neutrophil extracellular traps (NETs), tumors, liquid biopsy, metastasis, biomarkers, tumor microenvironment, immune system, NET inhibitors

## Abstract

Cancer remains a major global health challenge requiring the development of diagnostic and therapeutic strategies. Liquid biopsy is considered a promising minimally invasive tool for cancer screening, prognosis and treatment monitoring. Recent studies suggest that neutrophil extracellular traps (NETs) may also be potential liquid biopsy markers. NETs are web-like chromatin structures released by neutrophils in response to various stimuli to trap and neutralize pathogens. However, excessive or dysregulated NET formation has been implicated in tumor progression and metastasis. Elevated levels of NETs have been observed in patients with various types of cancer and correlate with disease stage and prognosis. The presence of NET markers such as citrullinated histone H3 (H3Cit), neutrophil elastase (NE) and myeloperoxidase (MPO) has been associated with higher tumor burden and poorer clinical outcomes. Several studies have shown a positive correlation between NET markers and circulating free DNA (cfDNA) levels, suggesting that NETs may increase the sensitivity of liquid biopsy in detecting and monitoring cancer progression. This review examines the role of NETs in the tumor microenvironment, their contribution to cancer progression and metastasis, and their potential use in liquid biopsy and cancer therapy.

## 1. Introduction

Cancer remains a significant challenge to modern medicine, with millions of new cases and deaths reported globally each year, as shown by the Global Cancer Observatory [1]. This highlights the urgent need for effective and quick diagnostic and monitoring tools for early detection, accurate staging and personalized treatment. Liquid biopsy is a minimally invasive approach that allows disease detection in peripheral blood samples, emerging as a valuable avenue for cancer management, advancing prognostics and treatment selection and monitoring. This method is based on detecting, isolating and characterizing blood elements such as circulating tumor cells (CTCs), circulating free DNA (cfDNA), exosomes and tumor-educated platelets (TEPs) and circulating mRNA (circ-mRNA).

CTCs are cells that detach from the primary tumor and enter the bloodstream, potentially leading to metastasis. Detecting even a single CTC can indicate a developing cancer process, enabling faster treatment and increasing survival chances [2,3,4]. The role of CTCs in defining progression-free survival (PFS) and overall survival (OS) has been proven in several types of cancers [5,6,7,8,9,10]. However, the rarity of CTCs compared to other blood cells necessitates advanced techniques for their isolation and analysis [11,12,13,14,15]. cfDNA, another key component of liquid biopsy, is released by tumor cells into the bloodstream and carries information about tumor-specific genetic alterations. While cfDNA is rapidly degraded, it can still reflect ongoing changes in the tumor, correlating with tumor size, stage and the presence of metastases [16,17,18]. Analysis of cfDNA can provide valuable insights for diagnosis, monitoring treatment response and detecting minimal residual disease [19,20,21,22,23,24,25,26].

While few elements of liquid biopsy, including CTCs and cfDNA, have already been established as tumor markers, neutrophil extracellular traps (NETs) have recently gained attention as potential biomarkers. NETs are web-like structures released by neutrophils in response to various stimuli, primarily known for their role in host defense by trapping and killing pathogens. This process is primarily mediated by the release of antimicrobial proteins such as neutrophil elastase (NE), myeloperoxidase (MPO) and histones, which are embedded within the NET structure. These proteins act in concert to immobilize and destroy invading microbes, forming a physical barrier that prevents their spread and contributes to the overall immune response [27,28]. However, it is important to note that while NET formation is beneficial for combating infection, excessive or dysregulated NET release can contribute to various pathological conditions, including cancer [29,30]. This article will delve into the role of NETs in the tumor microenvironment, their contribution to cancer progression and metastasis, and their potential as a liquid biopsy marker.

## 2. NET Production

NETs are produced in the NETosis process, which exists in two distinct forms: classical (suicidal) NETosis, culminating in cell death, and vital NETosis, which preserves cell viability and function [31].

A 1996 study first described a unique form of neutrophil suicide, distinct from apoptosis or necrosis, triggered by phorbol 12-myristate 13-acetate (PMA). The authors described the three main stages of the process: gradual decondensation of chromatin, nucleus swelling and spilling of the nucleoplasm into the cytoplasm, and perforation of the membrane. However, the significance of this observation remained unrecognized until 2004, when NETs were first described. Brinkman et al. [29], using an in vitro system, demonstrated that PMA or interleukin-8 (IL-8) induced neutrophil suicide, releasing web-like structures of DNA decorated with elastase and histones. Subsequent research has identified a wide array of NET-inducing stimuli, including lipopolysaccharides, GM-CSF/C5a and IFN-α/IFN-γ/C5a [29,32,33,34,35]. Additionally, various stimuli can induce the formation of NETs, including several viruses [36], bacteria [37], anti-neutrophil cytoplasmic antibodies [38], fungi [39], activated platelets [40] and calcium ionophores [41,42]. Detailed in vitro cellular imaging has revealed that NET release is often an NADPH oxidase (NOX)-dependent cellular death process, characterized by nuclear envelope disintegration following chromatin decondensation, and the mixing of nucleic acids and granular proteins within a large intracellular vacuole. However, NOX-independent NET formation, associated with calcium influx and mitochondrial ROS production, has also been documented [43]. Ultimately, NETs are released via plasma membrane perforation and cell lysis.

**Figure 1:**
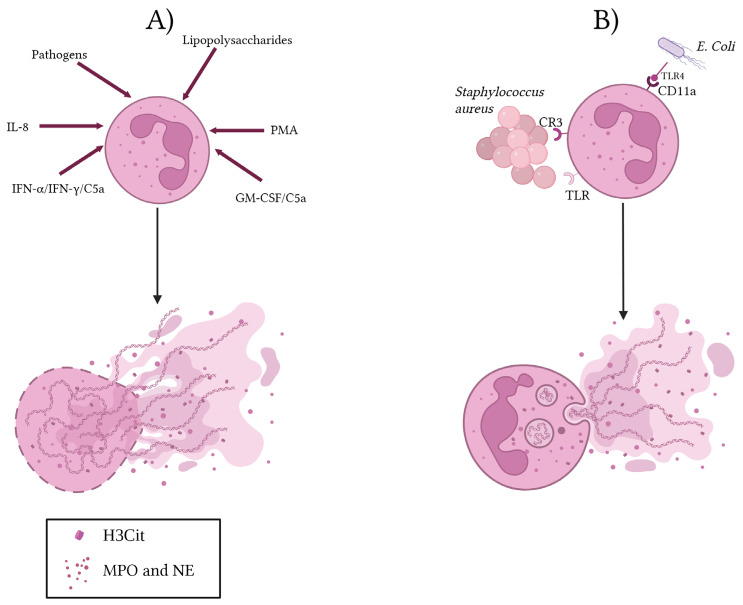
Suicidal (**A**) and vital (**B**) NETosis. Two types of NEToses are presented in the figure. (**A**) Classical (suicidal) NETosis, induced by pathogens, LPS, IL-8, PMA, IFN-α/IFN-γ/C5a and GM-CSF/C5a, leads to neutrophil death. (**B**) Vital NETosis, induced by Staphylococcus aureus via CR3/TLR and *E. coli* via TLR4/CD11a, allows neutrophils to remain viable. In this process, NET release does not cause neutrophil death. Created with www.BioRender.com (www.biorender.com, accessed on 30 June 2025).

In contrast, vital NETosis leads to the release of intracellular content without neutrophil lysis, as various research groups have described [32,34,44,45,46,47,48]. This process was initially observed in response to granulocyte–macrophage colony-stimulating factor (GM-CSF) stimulation, followed by C5a or LPS. NET formation occurred within a few minutes of stimulation and was associated with the release of mitochondrial DNA [32]. This rapid response was also observed following stimulation with Staphylococcus aureus in human and mouse neutrophils both in vitro and in vivo [44]. While Gram-positive bacteria like S. aureus induce NETs via complement receptor 3 (CR3) and Toll-like receptor (TLR) activation, vital NETosis triggered by Gram-negative bacteria requires TLR4 activation of platelets and subsequent direct neutrophil–platelet interaction via CD11a (Figure 1).

ApoNETosis is a recently identified process of NET formation that combines elements of both apoptosis and NETosis. Unlike typical apoptosis, necrosis, or other known forms of NEToses, ApoNETosis is specifically triggered by high-dose UV radiation. This unique pathway involves the activation of caspase 2, mitochondrial ROS production and NOX activity. Unlike other forms of NEToses, ApoNETosis does not involve histone citrullination, suggesting a lack of PAD4 enzyme activation and an increase in intracellular calcium concentration. While apoptosis is activated in ApoNETosis, the typical ’bubbling’ of the nucleus does not occur; instead, the DNA undergoes decompression and forms NET networks, as in NETosis. This process requires transcriptional activity and the activation of p38 MAPK kinase. The discovery of ApoNETosis has significant implications for UV therapies in autoimmune skin diseases like psoriasis and vitiligo. A deeper understanding of this mechanism may lead to improved therapies, enhancing efficacy and safety [49,50,51,52,53,54,55,56,57,58,59,60,61,62].

## 3. NETs in Cancer

NETs have been implicated in cancer progression and metastatic dissemination in human tumors. Elevated levels of NET-related components have been observed in the plasma of patients with various cancers, including lung, pancreatic and bladder cancer, compared to healthy controls [63]. Li et al. demonstrated the presence of NETs in lung tissues, peripheral blood, and sputum of lung cancer patients [64]. Similarly, in colorectal cancer patients, increased levels of NETs produced by neutrophils after in vitro stimulation were associated with poor clinical outcomes [65,66].

A prospective analysis conducted on 45 plasma samples from female patients newly diagnosed with breast cancer highlights an association between NET levels and disease stage [67]. NE-DNA complexes were assessed using ELISA, with optical density dichotomized at the median for comparison (categorized as low and high NE-DNA levels). Increased NE-DNA complexes were observed in regional and metastatic disease, supporting the proposed mechanism linking cancer progression and metastasis to NET formation [67]. Cancers significantly impact the life cycle of neutrophils, altering their capacity to form NETs. Cytokines and chemokines, such as G-CSF, CXCL1, IL-6, IL-1β, IFN-γ and TNF-α, which are secreted by cancer cells, intensify emergency granulopoiesis. This results in an increased presence of both mature and immature neutrophils in the circulation. Mature cells demonstrate a greater capacity for NETogenesis than their immature counterparts. Neutrophil activation occurs through receptors such as CXCR2, CXCR4, CD11b, TLR4 and RAGE, which respond to chemokines and recognize stress signals and DAMPs, thereby facilitating migration. NETogenesis can be initiated intracellularly through ROS production, PAD4 activation and ERK/MAPK pathways. The ability to form NETs is also influenced by age-related factors, such as clonal hematopoiesis (CHIP), or mutations in genes such as *JAK2*, which promote tumor progression and thrombotic complications. The epigenetic and metabolic reprogramming of bone marrow progenitors in the ’trained immunity’ mechanism can increase NETogenesis in the long term, acting either as anti-tumor or pro-tumor depending on the inflammatory context [68,69,70,71,72,73,74,75,76,77,78,79,80,81,82,83,84,85,86,87,88,89,90,91,92,93,94,95,96,97,98,99,100,101,102,103,104,105,106,107].

Hypercoagulable state present among patients with gastric cancer (GC) results in higher morbidity and mortality. A lot of studies have shown that NETs play an important role in thrombus initiation and progression. Yang et al. [108] suggest that procoagulant NETs are released by neutrophils because they are primed via a systematic environment created by gastric cancer. They examined 48 patients with GC and 36 healthy ones. By using immunofluorescence microscopy and ELISA technique, it was confirmed that neutrophils from GC patients release significantly more NETs than healthy controls. Also, the plasma from GC patients induced the neutrophils isolated from the control group to release NETs, and NETs released by GC neutrophils increased the potency of plasma from healthy controls to generate fibrin and thrombin [109].

**Figure 2:**
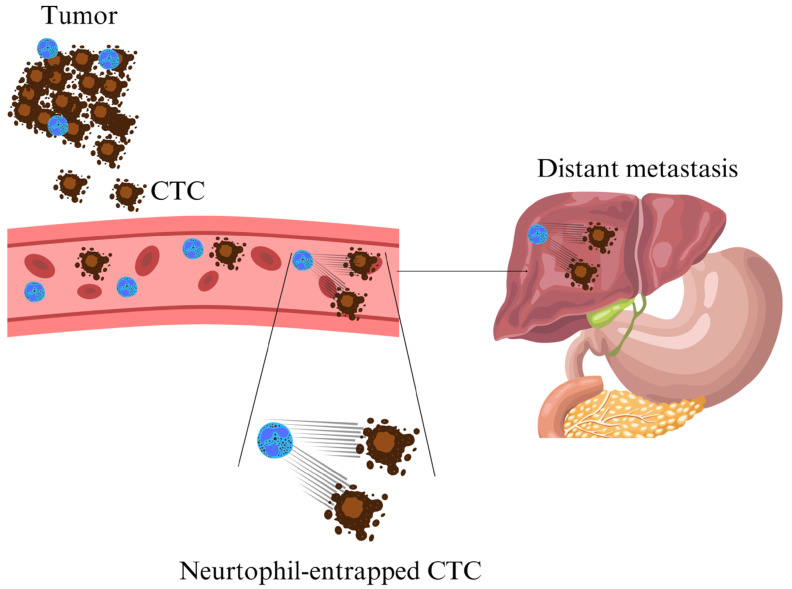
The role of tumor-associated neutrophils (TANs) and NETs in tumor cell capture and metastasis: Schematic representation of neutrophil involvement in tumor metastasis, showing CTC capture and transport. Neutrophils, through entrapment, facilitate CTC migration to distant sites via NET formation, promoting metastatic niche development.

Additionally, in the absence of infection, cancer cells were observed to promote NETosis in vivo and in vitro [110,111]. Additionally, tumor-associated neutrophils (TANs), a significant component of the tumor microenvironment, are strongly linked to NET formation [112,113]. Generally, TANs are considered pro-tumorigenic, with tumor-derived factors driving their recruitment, activation and differentiation within the tumor microenvironment [114]. Notably, TANs can re-enter circulation and capture CTCs by triggering NET release [115]. These NETs then foster the captured tumor cells’ invasion and migration to distant sites, effectively shielding them from cytotoxic lymphocytes. Furthermore, tumor-derived factors contribute to establishing pre-metastatic niches, promoting neutrophil infiltration and increased NET deposition, thereby facilitating metastasis (Figure 2) [115].

### 3.1. NETs in Cancer Progression and Metastasis

NETs possess robust adhesive properties, allowing them to bind pathogens and platelets. Studies theorized that this adhesive nature supports intravascular support, promoting tumor cell adhesion and extravasation in hematopoietic metastasis [116]. The web-like structure and adhesive qualities of NETs allow them to capture CTCs, facilitating their dissemination in the bloodstream, thus aiding the metastatic process [117]. In a murine study model, Cools-Lartigue’s group suggests that NETs can trap CTCs analogously in the context of severe postoperative sepsis, promoting early adhesion of tumor cells to distant organ sites [118]. The expression of β1-integrin plays a key role in this CTC entrapment by NETs both in vitro and in vivo [119]. Najmeh et al. used an in vivo murine model of intra-abdominal sepsis to mimic the postoperative inflammatory environment and demonstrated that inflammation upregulates β1-integrin expression, increasing early cancer cell adhesion to NETs. This effect is stopped when mice are treated with DNase 1 [119]. This mechanism highlights the link between inflammation and metastasis, further emphasizing the role of NETs in cancer progression.

Beyond their role in capturing circulating tumor cells, NETs also influence the tumor microenvironment itself. Park et al., in murine models of breast cancer, demonstrated that tumors induced by metastatic cell lines recruit higher proportions of neutrophils at the tumor site compared to tumors induced by non-metastatic cell lines [110]. Intravenous administration of metastatic cell lines leads to NET deposition in the lungs, fostering the formation of a metastatic niche even without systemic inflammation. Adding another layer of complexity, Yang et al. elucidated a novel mechanism by which neutrophil DNA and NETs promote cancer metastasis [120]. Cancer cells introduced into the lungs of murine models trigger neutrophil recruitment and NET formation, showing how cancer cells themselves have the potential to induce NET formation. Furthermore, the transmembrane protein CCDC25, expressed by breast cancer cells, can detect distant NETs and guide cancer cells toward them. Neutralizing antibodies against CCDC25 reduced NET-mediated metastasis, revealing its potential as a therapeutic target.

In addition to these mechanisms, NETs can promote metastasis through endothelial damage, triggering additional inflammation. This activation subsequently stimulates platelets and the remaining neutrophils, leading to further NET release. Platelet activation induced by NETs can worsen several adverse consequences linked with advanced metastatic breast cancer, such as venous thromboembolism (VTE) [121,122]. An excess of NETs overwhelms the phagocytic capacity of endothelial cells against NETs. It promotes vascular leakage and endothelial-to-mesenchymal transition through the degradation of VE-cadherin and the further activation of β-catenin signaling. This facilitates CTC adhesion to activated endothelium, extravasation, and the establishment of new metastatic niches [123].

### 3.2. NET Protection of Cancer Cells

Research has shown that chemokine receptor agonists CXCR1 and CXCR2, produced by tumor cells, induce the formation of NETs, which act as a shield protecting tumor cells against cytotoxicity mediated by natural killer (NK) cells and T cells. This was initially observed in vitro, where supernatants from various primary melanoma and colon carcinoma cell lines induced NET formation in neutrophils from healthy volunteers and polymorphonuclear myeloid-derived suppressor cells (PMN-MDSCs) from cancer patients [124]. This effect was inhibited by blocking CXCR1 and CXCR2 using reparixin, a CXCR1 and CXCR2 antagonist, or a CXCR1-blocking monoclonal antibody [125]. Similarly, in vivo spleen PMN-MDSCs from mice bearing 4T1 breast cancer cells also underwent NET formation in response to CXCR1/CXCR2 chemokines.

Further experiments demonstrated the presence of NETs in 4T1 tumors, where H3 histone co-localized with dsDNA in neutrophil-positive areas within the cancers [110]. Treatment with reparixin reduced the in vivo presence of NETs, as confirmed in the Lewis lung carcinoma experimental model. Time-lapse confocal and intravital microscopy confirmed that NET-coated tumor cells evade direct contact with cytotoxic immune cells in both co-culture and subcutaneous cancer models [126]. This shielding mechanism, as observed by Teijeira et al., effectively prevents CD8+ T cell and NK cell-mediated tumor cell killing. Beyond physical barriers, NETs may also exert immunosuppressive effects through neutrophil-derived mediators that negatively impact CTL and NK cell function. Pharmacological inhibition of NET formation, using agents like GSK484, DNase I, or reparixin, significantly reduced tumor growth and metastasis. Importantly, these inhibitors synergized with combined immunotherapy (anti-PD-1 and anti-CTLA-4 monoclonal antibodies), revealing a promising strategy for enhancing cancer therapy [127,128,129,130].

### 3.3. Prognostic Role of NET Markers in Cancer

Growing evidence indicates that cancer patients exhibit elevated levels of NET markers compared to healthy individuals, which often correlates with poorer patient survival [65,108,131]. Studies have investigated NET release by stimulating neutrophils from cancer patients in vitro and measuring circulating NET markers in serum or plasma. However, the lack of standardized assays and specific antibodies targeting NET epitopes poses a challenge [132,133].

Despite these challenges, research has shown that NET formation induced by stimuli such as LPS and IL-8/CXCL8 is heightened in gastric [108] and colorectal [65] cancer. For example, in colorectal cancer patients, elevated preoperative levels of cfDNA were associated with the persistent disease one year after resection [134], while in breast cancer patients, cfDNA levels correlated with tumor characteristics such as size, nodal involvement, and clinical stage [134]. It is important to note, however, that increased cfDNA may also originate from sources other than NET formation, such as apoptotic or necrotic cancer cells, adding complexity to the interpretation of these findings.

Some studies suggest that markers more specific to NET formation, such as circulating MPO/DNA or NE/DNA complexes, as well as plasma levels of citrullinated histone H3 (H3Cit), are associated with poor prognosis in cancer patients [135]. Indeed, high levels of H3Cit have been linked to various cancers and correlated with neutrophil activation markers [136]. Moreover, elevated H3Cit levels were found to be independent prognostic factors for short-term survival in cancer patients [131]. Furthermore, in pancreatic adenocarcinoma patients, circulating DNA and MPO-DNA levels were associated with the clinical stage of the disease. Adding to the evidence for the role of NETs in cancer progression, neutrophil and NET markers like MPO and H3Cit have been detected in metastatic lesions of breast cancer patients, suggesting a potential role in metastasis [120]. However, caution is warranted in interpreting these findings, as the analysis of MPO-DNA complexes may not be specific to NET formation [132].

While circulating NET markers hold promise in predicting cancer patient outcomes, further studies and technical advancements are necessary to establish their prognostic and predictive utility across different cancer types and stages. Standardized definitions of threshold levels for different NET markers are needed to distinguish normal from pathological states [130]. Beyond simply measuring individual markers, the use of algorithms may also enhance prediction accuracy. A study by Zhang et al., involving 3298 NSCLC patients, grouped the patients into different NETosis subtypes using The ConsensusClusterPlus package and utilized the Enet algorithm to construct the NETosis Risk Score (NETRS) [137]. NETRS demonstrated a profound correlation with the clinical features of patients diagnosed with lung adenocarcinoma (LUAD) and demonstrated distinct prognostic disparities among different NETRS classifications. Importantly, significant differences emerged in prognosis, TME characteristics, immune-related molecule expression levels, gene mutations frequencies, immunotherapy response, and drug sensitivity. NETRS outperformed 20 previously published gene signatures in predicting LUAD patient survival and also proved valuable in predicting prognostic outcomes for all NSCLC patients across nine independent cohorts, highlighting its broad applicability as a prognostic tool in lung cancer [137].

## 4. Correlations Between NET Markers and cfDNA

To establish NET markers as novel elements of liquid biopsy, their presence should be correlated with the presence of other elements of liquid biopsy. Several studies have investigated the connection between NET markers and the amount of cfDNA in oncology patients. Pastor et al. examined NET markers MPO and NE alongside cfDNA in 219 patients with metastatic colorectal cancer (mCRC) and in healthy individuals [138]. Both NET markers and cfDNA were elevated in mCRC patients, with a moderately strong positive correlation between MPO and cfDNA and a weaker positive correlation between NE and cfDNA. Supporting this notion, Pisareva et al. examined, among others, the presence of NET markers and cfDNA in the plasma of patients with COVID-19, systemic lupus erythematosus (SLE) patients and mCRC patients [139]. Again, mCRC patients showed higher concentrations of MPO, NE and cfDNA than healthy individuals, confirming the positive correlation between these markers. Beyond colorectal cancer, in GC, the concentration of NET markers (MPO-DNA, NE) and cfDNA was established. Firstly, the concentrations of these three markers were significantly elevated in cancer patients compared to healthy individuals. Moreover, as the disease progresses, the concentration of all three biomarkers increases proportionally [108]. This highlights the potential for NET markers to detect cancer and monitor its progression.

The concentration of another NET marker, H3Cit, was also examined and compared to the amount of cfDNA. Wang et al. examined H3Cit levels and then compared them with cfDNA concentrations in glioma, ovarian, colorectal and lung cancer patients [140]. The results showed elevated concentrations in the tumor groups compared to healthy individuals. Importantly, both H3Cit and cfDNA levels rose with the advancing clinical stage, and H3Cit concentration correlated with cfDNA concentration. This suggests that H3Cit, like other NET markers, may reflect tumor burden and disease activity. In ovarian cancer, the relationship between cfDNA and NET markers like H3Cit, NE and MPO has been further explored. Kim et al. found that all three biomarkers were elevated in patients with high-grade serous ovarian cancer (HGSOC), particularly in those with advanced disease (stages III–IV), and a positive correlation was observed between H3Cit, NE and cfDNA [141]. A similar study focusing on MPO instead of NE also found elevated levels in HGSOC patients, although the difference was not statistically significant [142]. This emphasizes the potential for a panel of NET markers to improve the diagnostic accuracy of liquid biopsies in ovarian cancer.

Interestingly, a study on thyroid cancer patients demonstrated elevated circulating levels of NET biomarkers and neutrophil-related mediators [143]. Notably, MPO-DNA complexes correlated with cfDNA, CitH3 and MPO levels, particularly in elderly patients and those with metastatic disease, suggesting their potential as a liquid biopsy marker. This highlights the potential for applying NET markers across various cancer types. Finally, Wannberg et al. provided further evidence for the association between H3Cit, NE and cfDNA in a cohort of 160 cancer patients with various cancer types [144]. Their findings of increased concentrations of all three markers compared to healthy individuals underscore the potential for these markers to be incorporated into broader liquid biopsy panels for cancer detection and monitoring.

**Figure 3:**
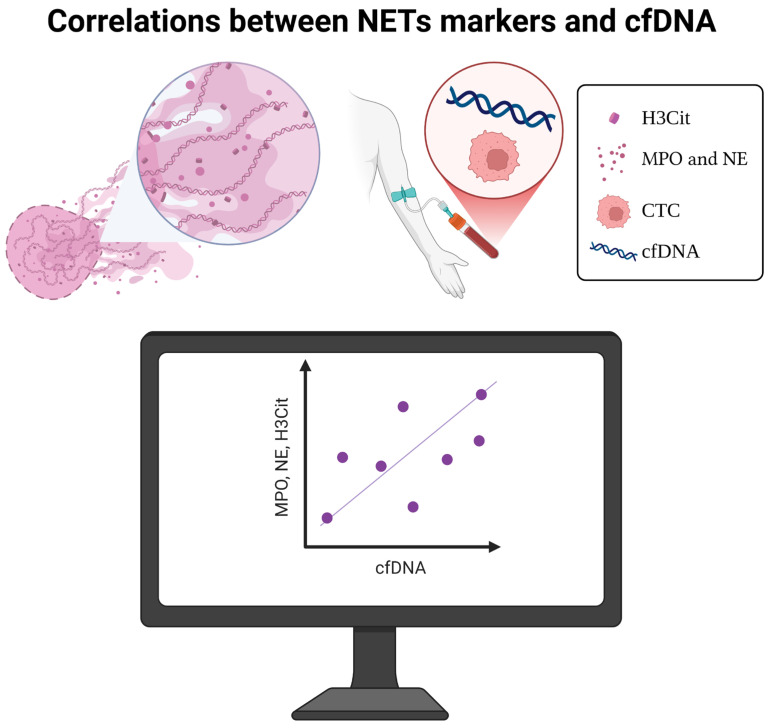
Correlation between NET markers and cfDNA levels in different cancer patients. The figure illustrates the proportional increase in NET markers (MPO, NE, MPO-DNA, H3Cit) and cfDNA across various cancers, including metastatic colorectal, gastric, glioma, ovarian, lung and thyroid cancers, highlighting their potential as liquid biopsy components for cancer detection and monitoring. Created with www.BioRender.com (www.biorender.com, accessed on 29 July 2025).

Taken together, these studies provide compelling evidence for the inclusion of NET markers in liquid biopsies. The consistent correlation between NET markers and cfDNA across various cancer types suggests that these markers reflect important aspects of tumor biology and may provide valuable clinical information (Figure 3).

## 5. NETs in Cancer Therapy

Predicting patient response to therapies targeting neutrophil extracellular traps (NETs) remains a significant challenge due to the lack of reliable markers. While markers like H3Cit and MPO-DNA may have prognostic value in cancer patients and indicate NET formation, their ability to predict treatment response is limited [131,145]. This is further complicated by the fact that studies correlating NET presence in tumor biopsies or GCSF expression with therapeutic response have not yielded conclusive evidence [110,146]. Furthermore, accurately identifying and quantifying NETs in body fluids using semi-quantitative methods like DNA-MPO association requires further investigation [66]. This highlights the need for more reliable methods to assess NETs and their role in cancer progression. Inhibiting NET formation is a promising cancer therapy due to its role in enhancing tumor metastasis. For instance, DNase treatment can degrade NETs and reduce metastases in animal models. Similarly, PAD4 and neutrophil elastase inhibitors can disrupt NET formation. Prostaglandins PGE1 and PGE2 can also downregulate NETosis via cAMP and calcium signaling, and drugs such as chloroquine can mitigate NET-related thrombosis. Furthermore, NET-targeting nanocarriers and markers such as H3Cit and MPO-DNA are under investigation for use in patient selection. Nevertheless, clinical application remains limited, and further research is required to validate these mechanisms in cancer therapy [42,46,66,88,110,118,119,131,145,146,147,148,149,150,151,152,153,154,155,156,157,158,159].

Despite these challenges, interest in the therapeutic potential of targeting NETs in cancer is growing. While experimental and clinical studies targeting NETs have mainly focused on non-cancer pathologies, such as autoimmune and pulmonary diseases, there is growing interest in exploring their therapeutic potential in cancer [46,66]. This stems from the hypothesis that NETs create a microenvironment promoting tumor cell dissemination and metastasis, suggesting that disrupting NETs could hinder these processes. Supporting this notion, Cools et al. demonstrated that disrupting NETs with DNAse or neutrophil elastase inhibitors (NEis) reduces adhesion and inhibits metastasis formation, highlighting NETs as a potential therapeutic target [160].

Given the potential of NET disruption as a therapeutic strategy, researchers are exploring various avenues for intervention. DNAse, for example, has a history of safe and effective use in humans with diseases like empyema and cystic fibrosis, administered intrapleurally or through inhalation, respectively [161,162,163,164]. Systemic administration in systemic lupus erythematosus (SLE) has been attempted without adverse effects [165]. Furthermore, numerous animal studies have demonstrated the anti-metastatic effects of DNAse 1 administration across various tumor models [166]. For instance, Patutina et al. observed a significant reduction in surface metastases in tumor-bearing mice treated with DNAse compared to controls in a murine model of LLC-induced pulmonary metastases [167]. Similarly, Sugihara et al. demonstrated reduced hepatic metastasis formation in a murine lymphoma model following DNAse1 administration, noting a decrease in intravascular arrest of neoplastic cells compared to untreated control mice [168]. These findings underscore the potential of DNAse as an anti-metastatic agent.

Beyond DNAse, other strategies for inhibiting NET formation or activity are being investigated. Inhibitors of molecules involved in NETosis, such as NE inhibitors and PAD4 inhibitors, currently used in non-cancer pathologies, could potentially improve the clinical outcome of cancer patients [153,158]. Additionally, physiologic substances like PGE2 and PGE1 appear to negatively regulate NETosis, suggesting their therapeutic potential in cancer treatment [42,148,154]. Chloroquine, a NET inhibitor, has been associated with reduced blood platelet aggregation and hypercoagulability in tumor-bearing mice and patients [66,159].

## 6. Conclusions

The urgent need for rapid cancer diagnosis and appropriate treatment selection drives the continuous improvement of diagnostic techniques and treatment monitoring methods. One of the key areas of interest is liquid biopsy. Based on the collected literature, it can be concluded that NET markers have the potential to become new elements of liquid biopsy. Their role in the cancer microenvironment, disease progression or protection of tumor cells warrants consideration as further tumor markers or potential therapeutic targets. Linking the levels of MPO, NE and H3Cit to the clinical stage of the tumor may lead to the conclusion that they can complement, for example, cfDNA and CTC analyses. This will provide a broader picture of the patient’s condition. However, further research is still needed for NETs to be widely introduced into clinical practice.

## Data Availability

Not applicable.

## Abbreviations

The following abbreviations are used in this manuscript:

NETs	Neutrophil Extracellular Traps
H3Cit	Citrullinated Histone H3
NE	Neutrophil Elastase
MPO	Myeloperoxidase
cfDNA	Circulating Free DNA
CTCs	Circulating Tumor Cells
TEPs	Tumor-Educated Platelets
circ-mRNA	Circulating mRNA
PFS	Progression-Free Survival
OS	Overall Survival
PMA	Phorbol 12-Myristate 13-Acetate
IL-8	Interleukin-8
GM-CSF	Granulocyte–Macrophage Colony-Stimulating Factor
IFN-α/IFN-γ	Interferon Alpha/Gamma
NOX	NADPH Oxidase
ROS	Reactive Oxygen Species
CR3	Complement Receptor 3
TLR/TLR4	Toll-like Receptor/Toll-like Receptor 4
CD11a	Cluster of Differentiation 11a
TANs	Tumor-Associated Neutrophils
ELISA	Enzyme-Linked Immunosorbent Assay
GC	Gastric Cancer
VTE	Venous Thromboembolism
VE-cadherin	Vascular Endothelial Cadherin
β-catenin	Beta-Catenin
CXCR1/CXCR2	C-X-C Motif Chemokine Receptor 1/2
NK cells	Natural Killer Cells
PMN-MDSCs	Polymorphonuclear Myeloid-Derived Suppressor Cells
CTL	Cytotoxic T Lymphocyte
PD-1/CTLA-4	Programmed Death-1/Cytotoxic T-Lymphocyte Antigen 4
NSCLC	Non-Small Cell Lung Cancer
LUAD	Lung Adenocarcinoma
TME	Tumor Microenvironment
NETRS	NETosis Risk Score
mCRC	Metastatic Colorectal Cancer
SLE	Systemic Lupus Erythematosus
HGSOC	High-Grade Serous Ovarian Cancer
G-CSF	Granulocyte Colony-Stimulating Factor
PAD4	Peptidylarginine Deiminase 4
PGE1/PGE2	Prostaglandin E1/E2
LLC	Lewis Lung Carcinoma
rhDNase	Recombinant Human DNase
DNAse	Deoxyribonuclease
